# Stable Field Emission from Single-Crystalline Zirconium Carbide Nanowires

**DOI:** 10.3390/nano14191567

**Published:** 2024-09-27

**Authors:** Yimeng Wu, Jie Tang, Shuai Tang, You-Hu Chen, Ta-Wei Chiu, Masaki Takeguchi, Lu-Chang Qin

**Affiliations:** 1 Research Center for Energy and Environmental Materials, National Institute for Materials Science, Tsukuba 305-0047, Ibaraki, Japan; wu.yimeng@nims.go.jp (Y.W.); chenyouhu@hotmail.com (Y.-H.C.); chiu.tawei@nims.go.jp (T.-W.C.); takeguchi.masaki@nims.go.jp (M.T.); 2 Graduate School of Pure and Applied Science, University of Tsukuba, Tsukuba 305-8577, Ibaraki, Japan; 3 State Key Laboratory of Optoelectronic Materials and Technologies, Guangdong Province Key Laboratory of Display Material and Technology, School of Electronics and Information Technology, Sun Yat-sen University, Guangzhou 510275, China; tangsh58@mail.sysu.edu.cn; 4 Department of Physics and Astronomy, University of North Carolina at Chapel Hill, Chapel Hill, NC 27599-3255, USA

**Keywords:** zirconium carbide, nanowire, electric field emission, chemical vapor deposition

## Abstract

The <100> oriented single-crystalline Zirconium Carbide (ZrC) nanowires were controllably synthesized on a graphite substrate by chemical vapor deposition (CVD) with optimized growth parameters involving Zirconium tetrachloride (ZrCl_4_), flow of methane (CH_4_), and growth temperature. The length of nanowires is above 10 µm while the diameter is smaller than 100 nm. A single ZrC nanowire was picked up and fixed on a tungsten tip for field emission measurement. After surface pretreatments, a sharpened and cleaned ZrC nanowire emitter showed a high emission current density of 1.1 × 10^10^ A m^−2^ at a low turn-on voltage of 440 V. The field emission is stable for 150 min with a fluctuation of 1.77%. This work provides an effective method for synthesizing and stabilizing single-crystalline ZrC nanowire emitters as an electron source for electron-beam applications.

## 1. Introduction

In the past decades, field emission has garnered increasing attention as a method to generate electron beams by applying a high electric field at room temperature, which extracts electrons through the surface of a metal or semiconductor via quantum tunneling [1,2]. Field emission electron sources have been developed and applied in microelectronic devices, electron microscopy, electron beam lithography, flat panel displays, and microwave devices due to their low energy consumption, narrow energy spread of emitted electrons, and long operational lifespan [3,4,5,6]. Additionally, numerous studies have focused on enhancing the electronic performance and controllability of the aforementioned devices at the nanoscale [7,8]. Lopes et al. achieved tunable electronic properties for carbon-based diodes and rectifiers by controlling the current rectification ratio and direction in ensemble molecular diodes (EMDs) through temperature regulation [9]. Shen et al. concentrated on modifying organic–inorganic systems via molecular doping, demonstrating that organic molecule doping significantly improves the performance of g-ZnO-based nano-electronic devices and broadens the tunable range of their field emission capabilities [10]. Nanowires with favorable geometry and controllable composition are considered promising nanostructures for cold field emission electron sources [11,12,13,14]. Various nanowires have been extensively researched for their potential to serve as stable cold field emission electron sources [15,16]. For example, Laurent et al. achieved a controllable current density of 1 mA cm^−2^ using a vertically aligned cobalt nanowire array [17]. Huang et al. demonstrated enhanced field emission characteristics through the fabrication of single In-doped ZnO nanowires [18]. Zhao et al. reviewed the synthesis methods of Si_3_N_4_ nanowires and their applications and prospects in optoelectronics [19]. 

Our team obtained a stable and controllable nanostructure of a one-dimensional LaB_6_ emitter with a low work function and achieved a high emission current density and low flicker noise without decay [20,21,22]. We focused on further developing this nanostructure using transition metal carbides, such as TiC, ZrC, HfC, and TaC, to achieve long-term stability at high current levels and enable its practical application in microelectronic devices [23,24,25]. Tang et al. demonstrated successfully the feasibility of using a single HfC nanowire as an electron source, owing to its low work function, high melting temperature, and outstanding chemical stability as a refractory ceramic material [24]. In addition to the aforementioned advantages, ZrC is also considered a potential material for future field emission applications due to its lower fabrication costs and safer production environment. Makie et al. obtained ZrC single crystals by arc floating zone refinement from sintered stock, which was then applied as a field emission emitter. Their work demonstrated the ability of ZrC emitters to operate under pressures far above those commonly found for field emission cathodes in the 1980s [26]. Recently, Chiu et al. demonstrated the feasibility of a ZrC single-crystalline nanowire as a field emission emitter, which showed a high field enhancement factor in their field emission measurements [23]. The nanowire tips with high crystallinity, clean surface, and low curvature can improve effectively their field emission performance. However, emission current stability and cathode reliability remained unsatisfactory when ZrC nanowires were utilized as field emitters [18]. In practical applications such as electron microscopy and electron beam lithography, stable field emission sources are essential for achieving precise imaging or etching [27,28]. An unstable electron beam current can lead to blurry images and poor etching precision, affecting directly the device’s efficiency and reducing its reliability and reproducibility [29,30]. Furthermore, the stability of the electron emission source is crucial for the long-term operation of the equipment. Significant current fluctuations may shorten the lifespan of the device and increase maintenance costs [30,31,32].

In this work, we focus on developing and utilizing optimized parameters of chemical vapor deposition (CVD) to synthesize <100> oriented single-crystalline ZrC nanowires and stabilize them for the characterization of their field emission performance. We optimize the crystallinity and morphology of the grown ZrC nanowires to improve their reliability as a cathode by adjusting several reaction parameters, including temperature, CH_4_ flow rate, and amount of ZrCl_4_ [33]. After a ZrC nanowire was assembled as a field emitter, surface pretreatment was applied to remove the irregular surface and oxidized layer from the ZrC nanowire tip. At the extraction voltage of 440 V, an emission current of density as high as 1.1 × 10^10^ A m^−2^ was achieved. Our single-crystalline ZrC nanowire emitter can emit stably for 2.5 h with a fluctuation of 1.77% in measurement. The results further confirm the practical potential and feasibility of the ZrC nanowires in field emission applications.

## 2. Experimental

In our synthesis experiment, ZrC structures were grown on a graphite substrate by simultaneously introducing Zirconium tetrachloride (ZrCl_4_) and methane (CH_4_) in the presence of nickel (Ni) nanoparticles as catalysts. The graphite substrate was designed as a rectangular sheet with dimensions of 8 × 15 mm. Single-crystalline ZrC nanowires were synthesized by using the CVD method. After the synthetic reactions were completed, a high density of ZrC nanowires was grown on the substrate. The chemical composition of the ZrC nanowires was characterized by X-ray diffraction (XRD; SmartLab, Rigaku, Tokyo, Japan) with Cu-K_α_ radiation. Scanning electron microscopy (SEM; JSM-6500F, JEOL, Tokyo, Japan) and transmission electron microscopy (TEM; JEM-2100F, JEOL, Tokyo, Japan) were used to characterize the microstructure of the grown nanowires. 

To measure its field emission characteristics, a single ZrC nanowire with a smooth surface and optimal aspect ratio was picked up and attached to a tungsten (W) needle tip, which featured a pre-prepared planar platform cut by focused ion beam milling (FIB; JFIB-2300, JEOL, Tokyo, Japan). Subsequently, carbon was deposited at the contact point between the ZrC nanowire and the W needle via electron beam-induced deposition, ensuring the stability and reliability of the ZrC emitter. The field emission measurements and surface pretreatments were applied in a high vacuum chamber with a pressure of less than 1 × 10^−7^ Pa.

## 3. Results and Discussion

**Figure 1:**
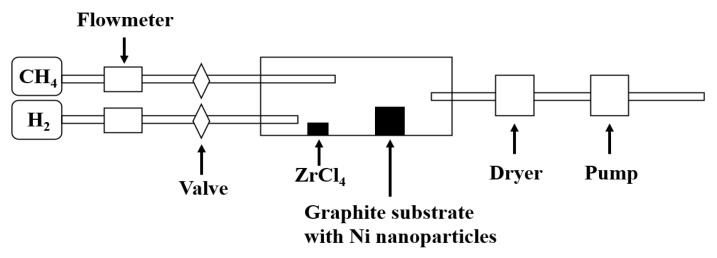
Schematic of the experimental setup for synthesizing ZrC nanowires.

The CVD synthesis system of our ZrC nanowires comprises a reactant controller at the front, a reaction zone, and a vacuum pump, as illustrated in Figure 1. In the synthesis experiment, the reaction is conducted in a quartz tube under a vacuum of less than 10^−1^ Pa. As the temperature increased, ZrCl_4_ powders (99.95% purity) with a melting point of 437 °C, placed in the low-temperature zone at the front end of the quartz tube, began to evaporate. The hydrogen (H_2_) gas flow carried the ZrCl_4_ vapors into the high-temperature reaction zone located in the center of the quartz tube. Then, CH_4_ gas was introduced into the reaction zone as a reactant. Subsequently, the two gases reacted for 20 min, and ZrC nanowires grew on a graphite substrate that was pre-coated with nickel (Ni) nanoparticles as a catalyst with a diameter of a few tens of nanometers. Graphite was chosen to minimize potential contamination at high temperatures due to the corrosive nature of HCl gas. In the preparation of Ni catalyst, 2 mg of Ni nanoparticles were added to 1 mL of alcohol, and 5 drops of the mixture were deposited onto the graphite substrate. Maintaining a high temperature for 20 min was found to be sufficient for obtaining the best results. Extending the reaction time would increase the dimensions and length of the ZrC nanowires but would not result in the higher density of nanowires [34].

In order to synthesize ZrC nanowires with a high aspect ratio and smooth morphology, we conducted extensive preparation experiments to determine the optimal parameter range. Experimental temperatures ranged from 1220 to 1310 °C (in 30 °C intervals), ZrCl_4_ amounts from 0.6 to 1.0 g (in 0.1 g increments), and CH_4_ flow rates from 60 to 120 mL min^−1^ (in 20 mL min^−1^ intervals). Each set of parameter combinations was repeated three times to ensure reproducibility. Table 1 lists several sets of parameter combinations used in the comparative experiments discussed below, discussing the effects of different parameters on the ZrC nanowires.

**Figure 2:**
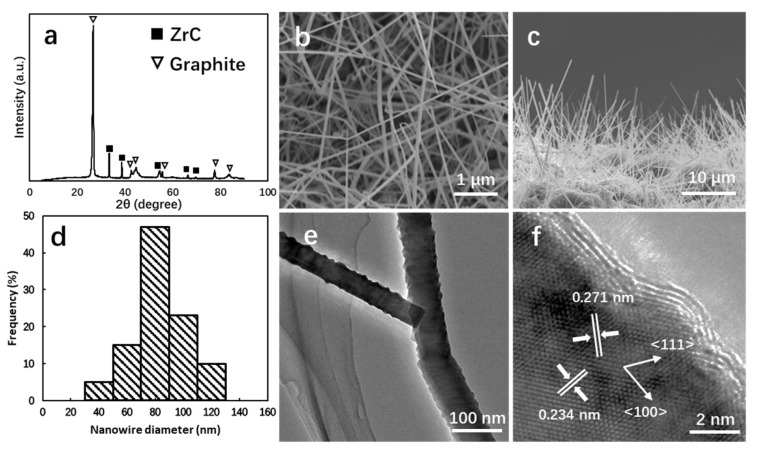
(**a**) XRD patterns display Bragg reflection originating from ZrC and graphite substrate. (**b**) The top-view and (**c**) tilt-view SEM images of ZrC nanowires show homogeneous growth. (**d**) Diameter distribution of ZrC nanowires. (**e**) TEM and (**f**) HRTEM image indicated that a single-crystalline ZrC nanowire grown in <100> direction.

Figure 2 shows the results of the structural characterization, including X-ray diffraction (Figure 2a), scanning electron microscopy (Figure 2b,c), transmission electron microscopy (Figure 2e,f), and the statistical plots of diameter distribution (Figure 2d) of one ZrC nanowire sample synthesized at 1280 °C by using 0.7 g of ZrCl_4_ and a CH_4_ flow rate of 100 mL min^−1^. The ZrCl_4_ vapors were carried to the reaction zone by the hydrogen (H_2_) gas at a flow rate of 1 L min^−1^ during the entirety of 20-min reaction. The reaction temperature of 1280 °C, slightly above the eutectic temperature of 1170 °C for the Ni-Zr alloy, ensures the precipitation of ZrC crystals during the reaction.

Figure 2a displays the X-ray diffractogram of the nanowires with graphite substrate. The XRD peaks are due to ZrC and graphite, corresponding to ICSD No. 01-089-2717 and ICSD No. 00-056-0160. The high purity of ZrC nanowires ensured no evidence indicating contaminations or impurities in the obtained sample. Figure 2b shows the top-view SEM images, revealing that the ZrC nanowires synthesized at 1280 °C exhibit a collimated morphology. The majority of nanowires are longer than 10 µm and have diameters of less than 100 nm. Figure 2d presents the diameter distribution of ZrC nanowires, based on SEM images taken from ten different locations. Over 80% of the nanowires have diameters below 100 nm, demonstrating the uniformity of the synthesis process. In the side-view image depicted in Figure 2c, more nanowires were observed to grow homogeneously and oriented at angles greater than 75 degrees relative to the graphite substrate at lower magnification. Further investigation of the nanowire structure and morphology using TEM is presented in Figure 2e,f. Figure 2e shows the typical morphology of a single nanowire with a diameter of 66 nm. A high-resolution transmission electron microscopy (HRTEM, JEM-2100F, JEOL, Tokyo, Japan) image of the same ZrC nanowire is displayed in Figure 2f. The surface of the nanowire is covered with a layer of thickness of 1–2 nm. The layer refers to graphite with a lattice spacing of 0.34 nm. These graphite layers, along with the oxide layer, can affect the field emission characteristics and will be removed during the pretreatment phase prior to testing. The lattice spacings measured inside from the HRTEM image are 0.271 nm and 0.234 nm, corresponding to the interplanar spacing of the (111) and (200) lattice planes of the ZrC crystal, respectively. These results indicate that the ZrC nanowire is a single crystal oriented in the <100> direction, grown uniformly on the graphite substrate.

**Figure 3:**
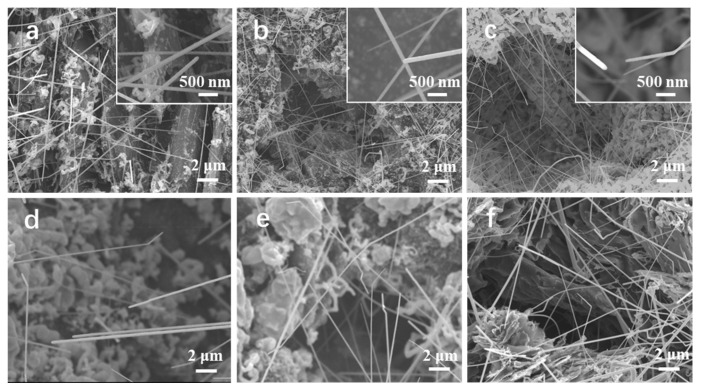
SEM image of ZrC nanowires synthesized with (**a**) 0.6 g; (**b**) 0.7 g; (**c**) 1.0 g of ZrCl_4_ at 1280 °C. SEM image of ZrC nanowires synthesized at 1280 °C with 0.7 g ZrCl_4_ and a CH_4_ flow rate of (**d**) 60 mL min^−1^; (**e**) 80 mL min^−1^; (**f**) 120 mL min^−1^.

To investigate the influence of ZrCl_4_ and CH_4_ as reactants on the growth of ZrC nanowires, a series of synthesis experiments were conducted by varying the amount of ZrCl_4_ and CH_4_ flow rate, respectively. Figure 3a shows the morphology of ZrC nanowires grown with 0.6 g ZrCl_4_ at 1280 °C. The ZrC nanowires synthesized on the substrate have diameters ranging from 50 to 150 nm. Additionally, most of the nanowires are aligned vertically and have smooth surfaces. Figure 3b,c show the SEM images of the ZrC nanowires when the amount of ZrCl_4_ was increased to 0.7 g and 1.0 g, respectively. The ZrC nanowires synthesized with a slightly increased amount of ZrCl_4_ (0.7 g) did not show significant changes, whereas those synthesized with a substantially increased amount of ZrCl_4_ (1.0 g) exhibited kinked morphologies. Figure 3d–f display the morphology of ZrC nanowires synthesized at 1280 °C by using a CH_4_ flow rate of 60 mL min^−1^, 80 mL min^−1^, and 120 mL min^−1^ while maintaining the amount of ZrCl_4_ and the H_2_ flow rate at 0.70 g and 1 L min^−1^, respectively. The length and growth density of the ZrC nanowires increased with increased CH_4_ flow rate. Similar to the phenomena observed in the experiments with the substantially increased amount of ZrCl_4_, higher flow rates of CH_4_ led to kink structures as shown in Figure 3f. These observations suggest that a greater amount of ZrCl_4_ and CH_4_ flow rate would accelerate the reactions and facilitate the growth of ZrC nanowires, resulting in denser nanowires on the graphite substrate with a higher aspect ratio.

**Figure 4:**
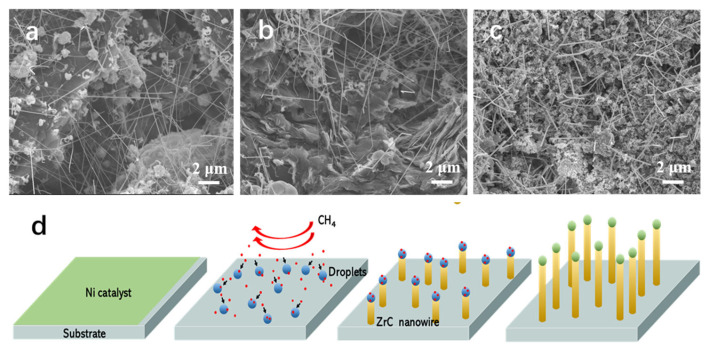
SEM image of ZrC nanowires synthesized with 0.70 g ZrCl_4_ at (**a**) 1220 °C; (**b**) 1250 °C; (**c**) 1310 °C. (**d**) Schematic of Vapor–liquid–solid (VLS) mechanism applied for ZrC nanowire growth.

Additionally, to prepare suitable ZrC nanowires with smooth surfaces and a high aspect ratio for field emission applications, we also investigated the effect of temperature on the morphology of ZrC nanowires synthesized by the CVD method. Figure 4a–c show the SEM images of ZrC nanowires synthesized at 1220 °C, 1250 °C, and 1310 °C, respectively, with the previously optimized ZrCl_4_ amount of 0.7 g and CH_4_ flow rate of 100 mL min^−1^. The ZrC nanowires synthesized at temperatures below 1280 °C exhibited much more kink structures, with a few even having multiple kinks. This phenomenon was also observed at a synthesis with the amount of ZrCl_4_ of 1.0 g and reduced temperatures in this set of experiments. Figure 4c shows that the ZrC nanowires synthesized at 1310 °C possess diameters exceeding 200 nm and exhibit rough and angular surfaces.

Our synthesis process follows the vapor–liquid–solid (VLS) mechanism, originally proposed by Wagner to account for whisker growth [35]. The VLS mechanism involves a catalytic liquid alloy phase capable of adsorbing rapidly vapors to supersaturation. Crystal growth proceeds from nucleated seeds at the liquid–solid interface. The synthesis relies on the following chemical reaction [23]

ZrCl_4_ + CH_4_ → ZrC + 4HCl (1)

and two partial reactions are proposed to describe this process

ZrCl_4_ + CH_4_ = Zr + C + 4HCl (2)

and

Zr + C = ZrC(3)

Figure 4d illustrates the whole growth process of ZrC nanowires on substrate. The intermediate product Zr formed alloys as droplets with Ni nanoparticles, which provided preferential nucleation sites for the formation and growth of ZrC on the substrate. The diameter of the ZrC nanowire is contingent upon the dimensions of the melting droplet. When the droplet reached the supersaturation, ZrC nuclei would start to form and grow sequentially layer by layer. Before the catalyst droplet contacts the ZrC nanowire body, an equilibrium between the catalyst droplet and the surrounding gas phase should already be established, as suggested by Schmidt et al. [36]. During the growth process, the surface tension of the droplet and the solid nanowire, as well as the interfacial tension between them, should remain in static equilibrium, ensuring effective synthesis of the nanowires during the CVD reactions. 

In our experiment, the higher amount of ZrCl_4_ or CH_4_ flow rate raised correspondingly the partial pressure of Zr/C vapor in the reaction zone. This variation could alter the composition of the melting droplet, disrupting the static equilibrium and causing changes in the droplet dimension and interfacial tension. These changes, in turn, could create new wetting conditions that convert horizontal interfacial tension into vertical forces, resulting in the accumulation of ZrC on the new growth interface and causing kinked structures of the ZrC nanowires. On the other hand, Zr-Ni alloy droplets formed initially at 1280 °C. When these droplets became supersaturated, ZrC nanowires would grow via layer-by-layer stacking. Providing more reactants would accelerate the incorporation of Zr/C into the Zr-Ni alloy droplets, thus speeding up the growth of ZrC nanowires. However, an excessively fast growth rate can also destabilize the growth of the ZrC nanowires. The catalyst droplets are more likely to shift at the nanowire tip to reach a new equilibrium under the new wetting conditions. Consequently, the nanowires would grow in a new direction, forming kinked structures. The oversupplied reactants would disrupt the static equilibrium between the surface tension of the catalyst droplet and the solid nanowire, resulting in kink structures. Similar principles apply to the effect of lowering temperatures. At a lower temperature, the suppressed rate of molecular thermal motion would disrupt the interfacial equilibrium, causing the nanowire to bend and grow in new directions. The greater thermal motion at high temperatures would force more residual product gas to precipitate at the solid–liquid interface, resulting in nanowires with lower aspect ratios. This epitaxial growth would also lead to the formation of jagged and rough surfaces.

**Figure 5:**
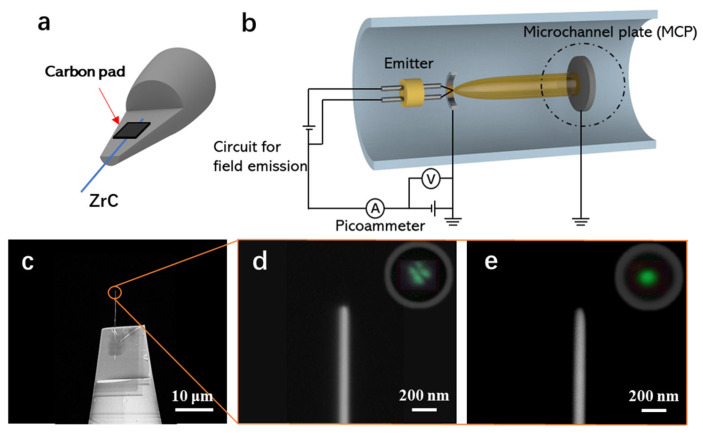
(**a**) Schematic of as assembled ZrC nanowire emitter. (**b**) A simplified schematic of our field emission measurement system. (**c**) SEM image of the assembled ZrC nanowire emitter. (**d**) Tip of ZrC nanowire before field evaporation pretreatment. The inset is the divergent field emission pattern on the MCP screen. (**e**) Tip of ZrC nanowire after field evaporation pretreatment. The inset displayed that the field emission pattern was concentrated at a single point on the MCP screen.

The assembly of a ZrC nanowire field emitter with high reliability is necessary to carry out field emission measurements. We used a nanomotor-driven probe to pick up one single ZrC nanowire and place it onto a W needle tip. All operations were monitored with an optical microscope, ensuring the selection of a ZrC nanowire with a smooth surface and high aspect ratio. The selected ZrC nanowire was synthesized at 1280 °C with 0.7 g ZrCl_4_ and CH_4_ flow rate of 100 mL min^−1^. Figure 5a shows a W needle was milled with FIB to furnish a platform for holding the ZrC nanowire. Subsequently, the ZrC nanowire was fixed onto the W platform by using carbon deposition to ensure the stability of the entire emitter. The carbon deposition was induced by an electron beam in an SEM. The SEM image of the as-assembled ZrC field emitter is shown in Figure 5c. As illustrated schematically in Figure 5b, the measurement system comprises several components, including an extractor, field emitter, microchannel plate (MCP), and a pico-ammeter. During the operation of field emission, a negative voltage is applied to the emitter to induce the emission of electrons from the nanowire tip. The MCP is set to collect the emission current and image the emission pattern.

When the ZrC nanowire emitter with surface adsorbates and impurities is placed into the high vacuum chamber (1 × 10^−7^ Pa) for the first time, the nanowire tip is usually a square shape, as shown in Figure 5d, and its field emission pattern is divergent, as shown in the inset of Figure 5d. Pretreatments are therefore required to ensure the field emission current was from the single-crystalline ZrC nanowire tip surface instead of contaminations and/or irregularities. A field evaporation was applied to an imaging gas with a positive and strong electric field, attracting the atoms to the tip. Since the electric field on the protrusion sites is strongest and the bonds between the adsorbates and the emitter are much weaker than the bonds between the Zr-C atoms, the contamination and the corner atoms will be evaporated first. After a few hops, the polarized atoms would ionize eventually and move toward the fluorescent screen. If the positive electric field is sufficient, atoms on the tip surface will be ionized and extracted. Subsequently, the strong electric field will furnish a clean and sharpened nanowire tip after field evaporation, as shown in Figure 5e. A stable field emission pattern from a rounded tip concentrated to a single point is shown in the inset of Figure 5e. Through a clean and sharpened nanowire tip, the electric field was highly concentrated at the center of the nanowire. Therefore, this field evaporation method can modify effectively the nanowire tip to improve its field emission characteristics.

**Figure 6:**
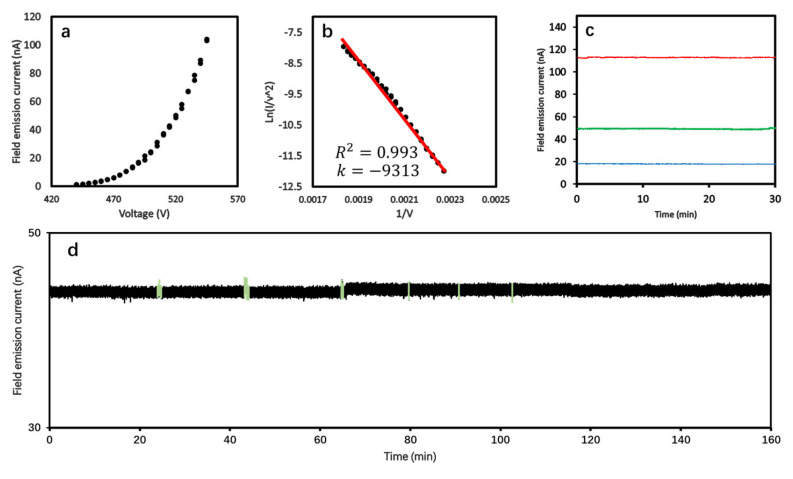
(**a**) I–V curve of field emission characterization. (**b**) F-N plot of field emission characterization. (**c**) The 30 min field emission stability of ZrC nanowire emitter under emission current of 18 nA, 49 nA, and 113 nA. (**d**) Long-term stability with a fluctuation of 1.77% in 2.5 h of measurement.

Figure 6a shows the correlation between the field-emission current (I) and extraction voltage (V) from the recorded I–V data. The turn-on voltage is 440 V with an emission current of 1.2 nA and the emission current reached 100 nA at 535 V. The field emission characteristics were evaluated by the Fowler–Nordheim (F-N) equation [37]

(4) $$J=1.54\times 10^{6}\frac{F^{2}}{\varphi }\exp\left(\frac{-6.83\times 10^{9}\varphi^{\frac{3}{2}}}{F}\right)$$

where J is the field emission current density, φ is the work function of the emission surface, and *F* is the electric field at the emitter tip. By using $J=\frac{I}{A}$ with *A* being the field emission area and F=βV with β being the field enhancement factor, which is determined by the local geometry of the electron emitter, a linearized relationship (F-N plot) is obtained

(5) $$\ln\left(\frac{I}{V^{2}}\right)=\ln(1.54\times 10^{6}\frac{A\beta^{2}}{\varphi })-6.83\times 10^{9}\frac{\varphi^{\frac{3}{2}}}{\beta V}$$

and

(6) $$k=6.83\times 10^{9}\frac{\varphi^{\frac{3}{2}}}{\beta }$$

is the intercept of the linear plot.

Figure 6b depicts the F-N plot of the ZrC nanowire emitter, exhibiting a highly linear relationship with an R^2^-coefficient of 0.993, thereby reinforcing that the field emission from the ZrC nanowire emitter conforms closely to the conventional cold field emission model described by the F-N equation. In addition, if we substitute the work function of ZrC (100) into the slope of the F-N plot, we can calculate the field enhancement factor *β* of the ZrC nanowire emitter, which is 4.62 × 10^6^ m^−1^, and a high emission current density of 1.1 × 10^10^ A m^−2^ was obtained. This *β* value is significantly higher than that of the commercial W needles, which is *β* = 1 × 10^6^ m^−1^, and it aligns closely with the empirical relationship *β* = 1/5*r*, where *r* is equal to 43.2 nm, representing the radius of curvature of the ZrC nanowire tip. This value is consistent with our observations from the SEM images of the assembled emitter with a tip radius of approximately 40 nm. The results further confirm the applicability of the empirical F-N equation at room temperature as reported in our earlier studies [20,22,23].

The stability of emission current is of essential importance for applications as electron sources. The most commonly used commercial cold field electron source, the W (310) filament, operates under an extremely high vacuum (EHV, lower than 10^−9^ Pa) to extract a usable emission current due to its major shortcoming: unstable field emission current that deteriorates rapidly. Figure 6c shows the measurements of the 30 min emission stability of our ZrC nanowire emitter at an emission current of 18 nA, 49 nA, and 113 nA, respectively, measured in a vacuum chamber operated in 1 × 10^−7^ Pa. The current stability, calculated by $\sum \left[{\left(I_{i}-\bar{I}\right)}^{2}\right]/\left[\left(n-1\right)\bar{I}\right]$ where *I_i_* (*i =* 1, 2, …, *n*) are the recorded emission currents, and the formula represents the variance in emission current divided by the average value of the current ($\bar{I}$), was 0.88%, 1.88%, and 1.10%, respectively. On the other hand, after the field emission current exceeded 40 nA, which is a value of practical importance, the ZrC nanowires maintained a stable emission current for over 2.5 h. Moreover, throughout the entire testing period, only six current values exhibited fluctuations exceeding 2%, as indicated by the light-colored region in Figure 6d.

## 4. Conclusions

In this work, single-crystalline ZrC nanowires with a high aspect ratio and smooth surface were synthesized using the CVD method by adjusting temperature, amount of ZrCl_4_, and CH_4_ flow rate. Additionally, the influence of these three parameters on the morphology of the nanowires was studied. We observed that an oversupply of reactants and lowered temperatures result in kink structures. A lower CH_4_ flow rate reduces significantly the growth density and length of the nanowires, while a higher reaction temperature results in nanowires with a smaller aspect ratio and rougher surfaces. Finally, the field emission characteristics of the ZrC nanowire emitter follow the conventional cold field emission model described by the Fowler–Nordheim equation. A high emission current density of 1.1 × 10^10^ A m^−2^ and field enhancement factor *β* of 4.62 × 10^6^ m^−1^ were obtained at a low extraction voltage of 440 V. The field emission current showed long-term stability with a fluctuation of 1.77% in 2.5 h of measurement. The successful development of ZrC nanowire emitters could offer additional options and support for research in hybrid molecular systems utilizing methods like molecular doping. This development extends the potential applications of these systems, thereby enhancing the scope of the findings and amplifying their relevance to the community of researchers and technologists. These results further demonstrate the practical potential and feasibility of the ZrC nanowires in high-performance electron beam technology applications.

## Figures and Tables

**Table 1:** Summary of the experimental parameters used in each experiment.

Case	ZrCl_4_ (g)	CH_4_ (mL min^−1^)	Temperature (°C)	Description
#1	0.6	100	1280	H_2_ gas with a flow rate of 1 L min^−1^ carried ZrCl_4_ vapors into the reaction zone.
	0.7			
	1.0			
#2	0.7	60	1280	CH_4_ gas was introduced last into the quartz tube, and the reaction was conducted for 20 min.
		80		
		120		
#3	0.7	100	1220	The heating rate of the reaction zone is 50 °C minute^−1^.
			1250	
			1310	

## Data Availability

The original contributions presented in the study are included in the article; further inquiries can be directed to the corresponding authors.
